# Topics of the Month

**Published:** 1895-09

**Authors:** 


### TOPICS OF THE MONTH.

Dr. William H. Daly, of Pittsburg, read a paper at the Hot Springs meeting of the Mississippi Valley Medical Association, in which he maintained the theory that malaria is a water-borne disease, taking the ground that the ingestion of water by the mouth would account for the majority of cases, to the exclusion of the generally accepted theory of aërial infection. Dr. Daly's observations have been made upon himself and upon several fellow-members of a sportsman's club, who have sought the marshes for duck shooting annually for the past twenty years. At first they braved the night air, relying on the free use of quinine as a preventive, but had drank the marsh water freely. Quinine having proved an inefficient safeguard, it occurred to Dr. Daly that it would be well to give up drinking marsh water. After testing this for a considerable time, he and his fellow-sportsmen found they could expose themselves freely to the night air of the marshes with impunity and without the aid of quinine. Dr. Daly extolled the value of Laverau's investigations as to the cause of malarial fever, and urged their more general teaching in the schools.

This subject is of much importance and deserves to be carefully investigated, and it is to be hoped that Dr. Daly and others will pursue the subject to a substantial conclusion.

